# Identifying and explaining the variability in development and implementation costs of disease management programs in the Netherlands

**DOI:** 10.1186/s12913-014-0518-0

**Published:** 2014-10-26

**Authors:** Apostolos Tsiachristas, Bethany Hipple Waters, Samantha A Adams, Roland Bal, Maureen PMM Rutten-van Mölken

**Affiliations:** Institute for Medical Technology Assessment, Erasmus University Rotterdam, P.O. Box 1738, 3000 DR Rotterdam, The Netherlands; Department of Health Policy and Management, Erasmus University Rotterdam, P.O. Box 1738, 3000 DR Rotterdam, The Netherlands; Center for Child and Adolescent Health Policy, Massachusetts General Hospital, Boston, Massachusetts USA

**Keywords:** Chronic disease, Cardiovascular risk, COPD, Disease management, Integrated care, Costs, The Netherlands, ICT

## Abstract

**Background:**

In the Netherlands, disease management programs (DMPs) are used to treat chronic diseases. Their aim is to improve care and to control the rising expenditures related to chronic diseases. A bundled payment was introduced to facilitate the implementation of DMPs. This payment is an all-inclusive price per patient per year for a pre-specified care package. However, it is unclear to which extent the costs of developing and implementing DMPs are included in this price. Consequently, the organizations providing DMPs bear financial risk because the development and implementation (D&I) costs may be substantial. The aim of this paper is to investigate the variability in and drivers of D&I costs among 22 DMPs and highlight characteristics that impact these.

**Methods:**

The data was analyzed using a mixed methods approach. Descriptive statistical analysis explored the variability in D&I costs as measured by a self-developed costing instrument and investigated the drivers. In addition, qualitative research, including document analysis and interviews, was conducted to explain the possible underlying reasons of cost variability.

**Results:**

The development costs varied from €5,891 to €274,783 and the implementation costs varied from €7,278 to €387,879 across DMPs. Personnel costs were the main component of development. Development costs were strongly correlated with the implementation costs (ρ = 0.55), development duration (ρ = 0.74), and number of FTEs dedicated DMP development. Organizations with large size and high level of care prior to the implementation of a DMP had relatively low development costs. These findings were in line with the cross-case qualitative comparison where programs with a longer history, more experienced project leadership, previously established ICT systems, and less complex patient populations had lower D&I costs.

**Conclusions:**

There is wide variation in D&I costs of DMPs, which is driven primarily by the duration of the development phase and the staff needed to develop and implement a DMP. These drivers are influenced by the attributes of the DMP, characteristics of the target population, project leadership, and ICT involved. There are indications of economies of scale and economies of scope, which may reduce D&I costs.

**Electronic supplementary material:**

The online version of this article (doi:10.1186/s12913-014-0518-0) contains supplementary material, which is available to authorized users.

## Background

In recent years, the healthcare community has been struggling to identify strategies to better manage the rise in the number of patients with chronic diseases. In the Netherlands, there has been a 17% growth in diagnoses of chronic disease and a 26% growth of patients with multiple chronic disease diagnoses in the past 8 years [1]. One possible method of managing the changes in healthcare that result from the increased diagnosis of chronic diseases is the D&I of disease management programs (DMPs), as has happened in the Netherlands. The Netherlands Organization for Health Research and Development (Nederlandse Organisatie voor Gezondheidsonderzoek en Zorginnovatie (ZonMw)) funded a research project to stimulate the implementation of DMPs and study their impact. Twenty-two healthcare delivery sites were awarded funding to participate in the study by developing and implementing a DMP; the majority of the sites were primary care cooperatives. In exchange for funding and support, each of the sites agreed to participate in research and put a DMP into place. Disease management was defined by the funding organization as:

a broad programmatic approach to chronic diseases, and a comprehensive care chain of diagnosis, treatment and counseling, as well as prevention, early detection and self-management. The approach is based on multidisciplinary care standards and is organized around the patient and his condition, as much as possible, in conjunction with his surroundings. (Call for proposals, page 7)

In the programs proposed by the care delivery organizations and in the literature about DMPs, patients’ participation in the treatment and management of their condition is a key component of DMPs [2], as is the involvement of multiple medical professionals in care planning and delivery [3], and the development and implementation of information systems (most frequently computer-based systems) to support chronic disease treatment and management [4]. The development of the DMPs at the selected sites included interventions altering the existing organization of healthcare delivery (e.g. regular multidisciplinary meetings and regular monitoring of patients) and interventions that were implemented as new processes (e.g. case management, self-management support, ICT). A more detailed description of the interventions is given in elsewhere [5].

In addition to funding for research on DMPs, the Netherlands has implemented a new financing system that impacted care for those with common chronic diseases (an overview of the Dutch healthcare system is provided in Additional file 1). In 2010, a bundled payment scheme was introduced in the Netherlands [6]. Bundled payment is a single payment that covers the multidisciplinary care required by a patient for one particular chronic disease during a predefined period of time [7]. The aim of this payment scheme was to improve the access, comprehensiveness, continuity, and other aspects of quality of care for chronic patients and to control the increasing expenditures for healthcare for patients with a chronic disease. In the first year of this funding reform, only care for diabetes mellitus (DM), chronic obstructive pulmonary disease (COPD), and cardiovascular risk (CVR) could be contracted in a bundled payment. Under the new payment scheme, chronic care is coordinated by groups of healthcare providers (called ‘care groups’). The bundled payment is negotiated between care groups and health insurers and includes 1) the costs of multiple caregivers in primary care (e.g. general practitioners, practice nurses, dieticians, physiotherapists, lifestyle counselor but not medicines, diagnostics and medical devices) as well as 2) the costs of care coordination, 3) information and communication technology (ICT), and 4) professional training and courses for healthcare providers. The latter three groups of costs can be seen as costs for the development and implementation (D&I) of DMPs.

Since DMPs involve a significant reorganization of healthcare delivery, they require substantial development costs (including but not limited to training costs, ICT costs, and costs of redesigning the care delivery process) and implementation costs (such as multidisciplinary team meetings, the costs of coordination between care-givers, the costs of monitoring and feedback). These costs are commonly carried by the organization that implements the program (i.e. care groups). To which extent the D&I costs of DMPs are included in the bundled payment is often unclear. This is despite recommendations to report these costs separately from the healthcare utilization costs and to include them in the price of implementing a DMP [8].

However, some insurers, including the largest one in the Netherlands, are not convinced about the benefits of bundled payment and do not provide this type of funding. Rather, these health insurers provide an add-on payment to cover the D&I costs, whereas the cost of healthcare is funded as before. It is not clear how this add-on payment is defined and to what extent it covers the D&I costs [9]. Considering this uncertainty and taking into account the substantial D&I costs of a DMP, care groups need to be able to correctly anticipate the D&I costs. Failing to do so could be financially disastrous for the providers of DMPs and serves as a disincentive for the implementation of DMPs.

The aim of this paper is to investigate the variability in and drivers of D&I costs among various DMPs and highlight characteristics of the DMPs that may explain the variability in costs during the project period.

### Study setting

The research for this paper was conducted as part of an evaluation of 22 Dutch DMPs spread across different regions of the Netherlands [5]. The DMPs were categorized in CVR (n = 9), COPD (n = 4), DM (n = 3), mental diseases (n = 3), and other (n = 3). The ‘other’ disease category includes DMPs for stroke, heart failure, and mix of CVR, COPD and DM. The Ethics Board of Erasmus University approved the data collection. All content has been anonymized.

## Methods

We used a mixed-methods approach to analyze data on D&I costs. To this end, we used descriptive statistical analysis to explore the variability in D&I costs, as well document analysis and interviews with project leaders, managers, and professional care givers.

### Quantitative methods

All development and implementation costs associated with the 22 DMPs were systematically collected. We developed a template that was based on the CostIt instrument of the World Health Organization (WHO) [10]. This template was completed during face-to-face interviews with DMP managers. During these interviews we also asked managers whether they had additional financing to cover the specific elements of disease management. The development costs included all costs made during the preparation phase of DMPs, e.g. labor costs for brainstorming sessions, training costs, and ICT support costs. The implementation costs included costs of multidisciplinary team meetings, coordination between care-givers, monitoring and feedback that occurred the year after the DMP implementation. We collected the development and implementation costs regardless of the budget holder for their financing; the budget holders could include care groups, health insurers, and/or government. The labor costs were calculated using the full-time equivalents (FTEs), duration of involvement in the project and the gross salary of medical, administrative, ICT, management and other personnel. Operating costs (including costs of professional courses, information/ communication, licenses, and materials) were calculated based on volumes and unit prices as stated in the template. Capital costs (such as building and purchase of ICT) were calculated based on their volume (for buildings that was square meters) and unit prices (for buildings that was Euro per square meter) and they were amortized over their lifespan as suggested by the WHO [10]. In the analysis, we included the development costs during the development phase, the annualized development costs, and the implementation costs in the year after implementation.

In addition to D&I costs, we also collected data about the duration of the development phase (in months), the number of patients participating in a DMP, the total FTEs available to the organization providing a DMP, and the FTEs dedicated to developing and implementing the DMP. The level of chronic care integration was also measured at the start of providing a DMP and a year later by using the Dutch translation of the Patient Assessment of Chronic Illness Care (PACIC) [11]. This questionnaire was distributed to participants of 19 DMPs (no data for the 3 mental disease DMPs was available). The mean PACIC value of the participants in each DMP was used in the analysis.

Descriptive statistics were used to investigate the variability in D&I costs among 22 DMPs. Pearson correlation coefficients and Spearman correlation coefficients were calculated for normally distributed and non-normally distributed variables, respectively. The normality was tested based on the Kolmogorov-Smirnoff test. We also performed an analysis of variance based on ANOVA and Kruskal-Wallis estimates to explore differences in the development and implementation costs among disease categories. We also performed an analysis of variance to investigate differences in D&I costs among different payment methods during the development and implementation phases. The payments were categorized in normal (e.g. for GPs this is a mixture of fee-for-service and capitation payment), normal plus add-on payment for D&I costs, and bundled payment. Considering the small number of observations (n = 22) we also looked into various associations using scatter plots and graphs.

### Qualitative methods

In order to understand how various characteristics may influence the costs associated with the D&I of DMPs, we examined how program plans ‘travel’ from the grant proposal to the D&I of the DMPs [9], as well as what actually happened during the D&I phases of the program by exploring and analyzing the multiplicity of D&I in practice [12]. This approach enabled us to gain a deeper knowledge of the activities implicit in DMPs, including activities that influence how programs develop, how programs use the provided finances, and how project teams overcame (or not) difficulties in the early stages of programs.

Document analysis was the first step of the qualitative data collection. The documents analyzed included the grant applications and project plans submitted by project leaders, the call for proposals (Diseasemanagement chronische ziekten), and care organization websites. The documents were analyzed inductively to gain a better understanding of the DMPs, project leaders, and care providers. The content of the documents informed the development of the interview guide, which focused on D&I of DMPs in practice.

Two in-depth case studies were selected for this paper, which highlight the different D&I costs in the DMPs; 15 interviews with project leaders and clinicians were conducted and used in the case studies presented in this manuscript. Questions about the history and contexts of the DMPs were asked. Interviews were digitally recorded and detailed notes and observations were also made during the interviews. Interviews were conducted in Dutch or English and ranged from 30 minutes to 90 minutes. Interviews were transcribed and coded into themes. Quotes were translated by a native English speaker; the translations were later confirmed by a native Dutch speaker.

To better understand the variability in costs from a mixed methods perspective, the primary economic researcher (AT) and the primary qualitative researcher (BHW) met regularly and jointly reviewed the data. The economic and qualitative data have been integrated iteratively, after consensus by all authors. This was done through frequent meetings between the first two authors and the rest of the authorship team.

### Ethics statement

The study protocol was approved by the ethics committee of the Erasmus University Medical Centre of Rotterdam (September 2009). For more details see Lemmens et al., 2011 [5].

## Results

Adequate understanding of complex policy structures and the impact of their change requires multiple types of information. Mixed-methods research facilitates this by combining qualitative and quantitative research methods in order to identify, decompose, analyze, and understand complexities in healthcare [13]. Our research found a large variability in D&I costs between the researched DMPs. We uncovered three common characteristics of the studied DMPs that may explain the variability in costs between programs. These characteristics include attributes of the interventions, ICT systems, and the experience of the project leaders. The history of the programs, including personnel time invested and ICT systems already in place, may also play an important role in the variability in costs and is an underlying characteristic.

### Variability in D&I costs and cost drivers

**Figure 1:**
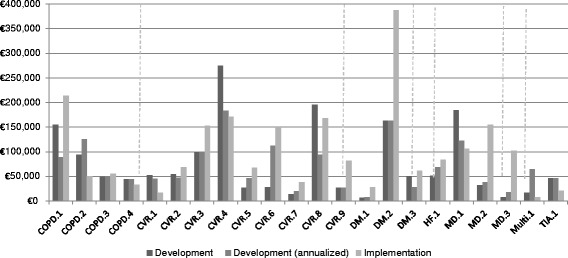
**Development and implementation costs per DMP.**

As Figure 1 shows, the development costs varied from €5,891 to €274,783 across DMPs and the implementation costs varied from €7,278 to €387,879 across DMPs. There was also large variation in D&I costs across DMPs in the same disease category. In some cases the development costs were higher than the implementation costs and in some other cases not. In addition, the four DMPs with the highest development costs among all DMPs also had the highest implementation costs. When annualized, the development costs varied also largely between and within disease categories and were also positively associated with the implementation costs.

**Figure 2:**
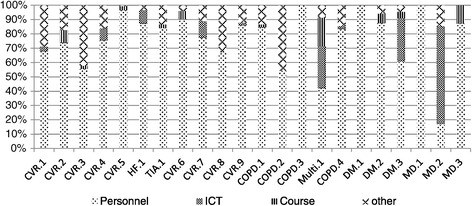
**Share of total development costs per cost component.**

Personnel costs were the primary component of development costs across 18 of 22 DMPs, accounting for more than 60% of total development costs (Figure 2). They were followed by ICT costs (maintenance and licensing) and the costs of professional courses as the main cost components of development costs.

The results from the descriptive statistical analysis are presented in Table 1. This table shows that our sample consisted of DMPs varying in the duration of the development phase (range: 3; 25 months), number of patients participating in the DMP (range 75; 3,400), total number of FTEs in an organization (range 1; 2,850), and number of FTEs involved in developing a DMP (range: 0.1; 2.5). The mean development costs were €75,832, the mean annualized development costs were €69,749 and the mean implementation costs were €100,827 across all 22 DMPs. The mean PACIC at implementation was 2.88 and a year later was 2.95. The Kolmogorov-Smirnoff test showed that the variables ‘number of DMP participants, ‘organization FTEs’, ‘development costs’ and ‘PACIC at implementation’ and ‘PACIC a year later’ were not normally distributed.

**Table 1:** **Descriptive statistics**

	**Mean**	**SD**	**Median**	**Min**	**Max**	**IQR**
Development duration (months)	12	6	12	3	25	6
Patients participating in DMP^#^	801	986	300	75	3,400	957
Organization FTEs^#^	433	841	33	1	2,850	256
DMP FTEs	0.76	0.58	0.60	0.10	2.50	0.63
Development costs^#^	75,832	72,727	49,972	5,891	274,783	85,917
Annualized development costs	69,749	47,807	48,141	7,855	198,188	66,704
Implementation costs	100,827	86,776	74,836	7,278	387,879	117,079
PACIC at baseline^#^ (1–5 best)	2.88	0.29	2.81	2.25	3.60	0.35
PACIC at year 1^#^ (1–5 best)	2.95	0.28	2.99	2.44	3.62	0.40

^#^The Kolmogorov-Smirnoff test rejected the assumption of normally distributed data; SD: standard deviation; min: minimum; max: maximum; IQR: interquartile range (Quartile 3-Quartile 1); FTE: full-time equivalent; implementation costs accrued within the first calendar year of DMP implementation.

The correlations between the D&I costs and potential cost drivers are presented in Table 2. Total development costs were strongly correlated with implementation costs (ρ = 0.55), development duration (ρ = 0.74), and number of FTEs dedicated to the development of a DMP (ρ = 0.54). The latter was found to be correlated also with the development duration (ρ = 0.49). The annualized development costs were correlated with the implementation costs (ρ = 0.65) and number of FTEs for the development of a DMP (ρ = 0.52). The results also showed a negative correlation between PACIC a year after implementation and development (ρ = −0.27) and implementation (ρ = −0.24) costs.

**Table 2:** **Correlation coefficients**

	**Development costs**	**Annualized development costs**	**Implementation costs**	**Development duration**	**DMP participants**	**Organization FTE’s**	**DMP FTE’s**	**PACIC baseline**	**PACIC year 1**
Development costs	1								
Annualized development costs	0.79 (0.000)	1							
Implementation costs	0.55 (0.008)	0.65 (0.001)^#^	1						
Development duration	0.74 (0.000)	0.24 (0.284)^#^	0.27 (0.228)^#^	1					
DMP participants	−0.12 (0.600)	0.02 (0.922)	−0.08 (0.707)	−0.09 (0.688)	1				
Organization FTE’s	−0.03 (0.887)	0.02 (0.940)	−0.00 (0.988)	−0.03 (0.880)	−0.14 (0.549)	1			
DMP FTE’s	0.54 (0.010)	0.52 (0.013)^#^	0.16 (0.482)^#^	0.49 (0.022)^#^	−0.04 (0.869)	−0.35 (0.110)	1		
PACIC baseline	−0.22 (0.366)	−0.02 (0.937)	−0.21 (0.388)	−0.24 (0.323)	0.21 (0.380)	−0.40 (0.095)	−0.29 (0.232)	1	
PACIC year 1	−0.27 (0.051)	−0.08 (0.049)	−0.24 (0.044)	−0.21 (0.396)	−0.23 (0.350)	−0.02 (0.932)	−0.28 (0.909)	0.64 (0.003)	1

^#^Based on Pearson correlations; FTE: full-time equivalent; in brackets are the p values of the correlation.

**Figure 3:**
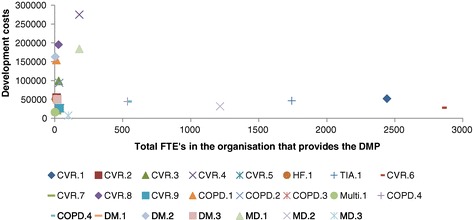
**Association between development costs and total number of FTEs in the organization.**

The relation between development costs and the total number of FTEs in the organization that provides a DMP is illustrated in Figure 3. This figure shows that large organizations had relatively low development costs compared to small organizations. This relation remained between the annualized development costs and the total number of FTEs in the organization that provides a DMP (see Additional file 2: Figure S4).

**Figure 4:**
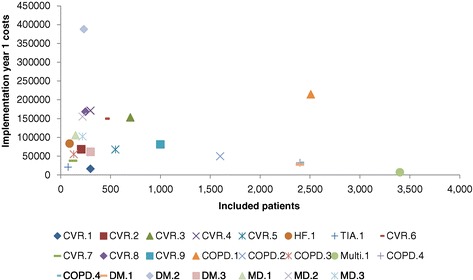
**Association between implementation costs and number of patients participating in a DMP.**

Figure 4 illustrates the relation between implementation costs and the number of patients participating in a DMP. We see that there might be a small negative relation between the two variables.

The results of the analysis of variance of D&I costs comparing different payment methods as well as different disease categories were not statistically significant. Figures illustrating these variances are presented in Additional file 2.

### Characteristics of the disease management programs

While each of the practice sites had a different method of addressing chronic disease through the funded DMP, the qualitative research uncovered characteristics that help to understand the differences in D&I costs. These characteristics include attributes of the DMP, ICT systems, and the experience of the project leadership teams. Qualitative data from two case studies will illustrate these characteristics. The diabetes case (DMP number 17) had relatively low D&I costs, while the CVRM case (DMP number 10) had high D&I costs.

### Case study: Diabetes (DMP number 17)

One of the project sites with a focus on adult-onset diabetes developed a DMP for clinicians and patients. The clinicians and project leaders at the site have invested time and effort in improving diabetes care for patients since 1999 by working in cooperation with the hospital and specialists, developing care protocols, and contracting with providers. Around 2006, the changes in diabetes care were formalised into an early form of disease management. The studied program was a continuation of a previously funded program. This site had two project leaders, the first of which was hired for the previously funded program to assist with the development of an electronic medical record and stayed on through most of the funded DMP. The first project leader was a professional healthcare consultant with a background in economics. The second (and current) project leader was the assistant of the first and took over the project leadership when the first project leader left, after a period of mentored transition.

#### Program attributes

For the disease management study, the project leaders worked with healthcare professionals to change how they thought about care and creating multidisciplinary care teams, implemented an updated networked electronic medical record system with a patient portal, and oversaw self-management education for patients.

The multidisciplinary care teams included nurse specialists, dieticians, general practitioners, nurses, chronic disease specialist assistants, ophthalmologists, podiatrists, and/or internists; members of the care team were located in multiple GP and specialist offices throughout the region. The care team worked together, communicating with each other frequently.

*I think that working closely with the dietitian especially… We sit together, we discuss a lot, and we can call the GP or practice nurse, and yes, several people are looking [at the case].* (Interview with diabetes specialist nurse)

Communication was seen as key to coordinating care. It was common for the staff, such as doctors, nurses, dieticians, and other clinical professionals, of disease management teams to sit together in a shared office and communicate about patients and care; however, this was not the only way that communication happened. The diabetes program had regular meetings with clinical professionals from multiples GP offices with the project leader overseeing the meetings. The project leader also sent out emails, reports, and posted information about the DMP online. Clinicians also communicated with one another through the networked electronic medical record. This system of coordinated care has been developed by investing in time for the project leader and clinician stakeholders over the years of the program.

One of the efforts of this disease management project focused on self-management through educating patients about diabetes. There were a variety of formal and informal educational opportunities for patients: group classes, clinical visits, and online. Patients could participate in voluntary classes, which were led by a nurse, doctor, and/or non-physician chronic disease specialist assistant. Clinicians were trained to lead by experts in diabetes education, an additional D&I cost-item which was hoped to be recouped in a reduced need for individual education sessions with patients. However, the classes were no longer offered as a result of lack of patient interest and attendance.

#### Internet-based communication systems

For the diabetes project, as is the case for many medical practices, the full development and use of an electronic medical record (EMR) was a complicated process. The GP offices involved in the diabetes project began working with software developers in 2006 to develop a more-limited version of an EMR. The project leaders, especially the first project leader, worked with clinicians and software developers to enhance the record. The newly enhanced record allowed for viewing the record by multiple clinicians (and the project leaders) at different physical locations, electronic referrals, and messaging between clinicians and between clinicians and patients.

Internet-based communication (ICT) systems, such as the EMR, have been seen as a remedy for the many predicaments in healthcare delivery and quality, one that promises more than is realized in most cases [14]. The implementation of ICT systems in healthcare delivery has been a lengthy process for project leaders and clinicians involved in the DMP, requiring planning, developing, implementing, and tailoring the system before the system can begin to meet the needs of clinicians and patients. At this site, the development of the ICT system began well before the program was funded as part of the study. By working with a previously developed system, this project leader and leadership team had the opportunity to gain the needed support from stakeholders, as well as work through the inevitable bugs and challenges in the system before the funded DMP officially began. These challenges included:

*The fact that the software builder couldn’t deliver what they said they would deliver. And still now we do not really have the perfect system and the perfect system does not exist, I know. But there are too many things that we want. But, there is no other software builder at this moment that DOES have it. The software builder itself has been in bankruptcy 2 times, once in 2007 and 2 months ago for the second time. Fortunately they worked together with another department who had been able to go on with the system, so that we were not cut off.* (Interview 1 with project leader 1)

As noted in the quote above, the investment of staff time and the hurdles that frequently occur in the early development phases of a networked electronic medical record occurred, in part, before the study and in the early phases of the study. Since the computer program had already been chosen, the time and effort needed to select a computer program and coordinate the program with existing record systems were not part of the study. As a result, much of the development work and growing pains of implementing an ICT system were not seen in the D&I costs for this aspect of the DMP.

One of the goals of the funded portion of the DMP was the D&I of a patient portal for the networked electronic medical record. While the project team was able to develop a patient portal in a timely manner, the development of the portal does not guarantee usage.

*Yes, we do have that but no-one uses it. We have talked and talked to get people to look, [telling patients] you have your own care dossier, your own plan, we can agree on goals there, you can report on how it is going, you can also tell us what does not go well, or if you have questions. Really easy, you can do that from your chair at home, you don’t have to come here if you don’t want to. But people don’t want that. It has cost money, because in order to offer the portal we had to expand our software package. Of the current 2700 people with diabetes I believe 15 now have a care plan.* (Interview 1 with second project leader, diabetes project)

While rates of patient participation in the portal were low, the numbers were expected to increase in the future as more internet-friendly patients are diagnosed. Though the usage remains low, this D&I cost is expected to have lasting impact well after the project period.

The networked electronic medical record has travelled from plan to action successfully in principle, in that the portal was developed in a timely and cost-effective manner, but not in the current day-to-day reality of the program, as very few patients used the portal at the time of the interview. This investment in future patients via a patient portal can be seen as a D&I cost. While the patient portal was successfully developed from a technical point of view, the implementation in practice will still require much effort on the part of the clinician, the project leader, and the expected future patients, who too must learn how to use the new system and whose time is frequently overlooked in accountings of D&I costs.

#### Project leadership

The cooperation hired an experienced project leader to oversee the diabetes DMP. Much like the groundwork done with the ICT system, the hiring of this first project leader occurred before the funding of the study. The project leader saw her role as giving support, both material and strategic:

*And I think that a unique thing is that what I do is I’m able to give support on the strategic thinking… where do you want to go to, what are the goals, what’s your mission, what’s the gain of it. And the second is how can we achieve that. So it’s not a consultant with only the advice but also what does it take to get there.* (Interview 1 with project leader 1)

The first and second project leaders led meetings, created reports from data extracted from the networked electronic medical record, and coordinated the efforts of the study team, such as sending out surveys. When the project leader resigned, she passed the role on to her assistant.

Effective leadership is crucial for bringing projects to life. In fact, in their seminal article on DMPs, Wagner et al. [3] point out that making the change needed for DMPs is “difficult, if not impossible, without strong leadership”. Leadership support was (and, in general, is) needed for multiple aspects of the DMP: for the successful implementation of a health ICT system [15], to guide the vision of the improvements in chronic care treatment and management [2,16], and to facilitate change in the healthcare delivery [17]. Yet project leadership skills and efficiency grow over time, as the project leader gains the trust of the clinicians, as the project leader is better able to understand the needs of the clinicians and patients, and as the project leader and the clinicians are able to adapt to one another.

### Case study: Risk of cardiovascular disease (DMP number 10)

A DMP conducted in two GP offices focused on improving care for those with an elevated risk of cardiovascular disease. The project team consisted of two GP-researchers (one of which served as a part-time project leader) and a nurse manager, who did the day-to-management of the program and study. As written in the grant proposal, the key elements of the program were*:*

- *a patient choice program to promote a commitment to the formulated treatment goals*
- *a focus on reaching people with a low socio-economic status (SES)*
- *the use of a web-based patient record* (Grant proposal)

This DMP was a newly formed project, developed by the project leader who had recently completed a Masters in Healthcare Management from a nearby university.

#### Program attributes

The disease management project focused on providing coordinated care with multiple clinicians to a challenging population: patients with an elevated cardiovascular risk and a lower SES.

*We have many patients, about 20% of the patients in the GPs practices are known to the GP as having one form of elevated cardiovascular risk. That’s a very big number of patients. Of those patients, about 8 or 9% are under regular control of the GP. And from those, a small part has a low SES. Especially patients at low SES do not follow our advice; you can see that as you look at the numbers. Most people, more people at low SES, dying of cardiovascular diseases, more people smoking… That’s the most important start of our project. And we don’t reach people with low SES, so we are looking at new methods of treatment of people with low SES.* (Interview 1 with project leader)

The patients with a lower SES were, and commonly are in healthcare, seen as a tricky population with multiple problems, less access to resources, and lower rates of literacy. Providing care and self-management education to this population was expected to be (and was) challenging for the project leadership team and clinicians, requiring a significant time investment. To be overcome, these challenges required effort on the part of the clinicians, patients, and project leaders.

Much of the investment of time for project leaders, in general, comes in the early stages of the DMPs. As time goes on in the course of programs, project leaders develop a better sense of the population and are better able to tailor attributes of the DMP for the needs of specific populations. This was the case in the CVRM program, as the project leader noted below:

*Then we ask the patient, do you want to look at your own patient file on the internet? And when he says yes, he can open his own file and see his own cardiovascular risk profile on the internet. Because we suppose that not every patient with low SES has a computer at home or can look at his file on the internet. So that’s why we ask this to every patient.* (Interview 1 with project leader)

This patient population required (more) time and effort from project leaders, clinicians, and medical office staff, as the patient population may not have had internet access, may have spoken limited Dutch, and may have had fewer economic and social resources for support. Accommodating this population to ensure good care required time during the clinical visit and, for the project leaders, time during the D&I of the DMP.

However, the challenges with the patient population were not the only challenges that the project leadership faced.

*Well, you’ve got to separate the problems: content level and organizational level. Content, I think, it actually runs smoothly. We must, of course, continue to develop, but that is going the way we want. Organizationally we have some problems. [Primary care] practices are (…) very large organizations now. So before we begin, we have to convince everyone of the importance of the research. That takes a lot of effort. Plus the implementation of such a project, in practice is not simple because practices are large organizations where 30 people work. Plus there are other members of the care group that need to be involved: the physiotherapist, the dietician.* (Interview with the project leadership team)

Effort and accommodation, in the form of meetings, telephone support, and emails, was needed to assist the clinicians in implementing the changes needed for implementing a DMP and for conducting research on the program.

#### Internet-based communication systems

As was seen in many other projects, the D&I of the networked electronic medical record required much time and effort over the course of years; this effort included working with outside vendors, outside educators, and outside funding agencies, as well as working with clinicians and GP office staff. The effort did not stop; changes and further tailoring continued after the record was in place. The project leaders and manager were key in these activities.

*And for the development of cardiovascular risk management, this is how far we are now: we have funding. We are now working with contract negotiations. And then we can start developing and the ICT supplier, if they are fast, can get us a beta version in three months’ time. We hope that we can really start with ICT in March, February… well, of course it is a problem to get financing. A negotiation problem. Yes, but we are happy that we have had luck.* (Interview with the project leadership team)

Because health insurers provided some of the financing needed for the D&I of the networked electronic medical record, there was much coordination work needed. Health insurers required extensive plans, budgets and presentations before financing was awarded. This was in addition to the work needed to develop the record, such as working with the developers and clinicians. Patient portals were included in the development of the networked electronic medical record, allowing patients to go online and access their record.

*You can see here, patients with active risk. This is what patients can see at home. The treatment goal of this patient was weight reduction of 6 kg in 3 months. And you can see at this point, he has reached a risk reduction of 80% of his goal. … Here is the plan and what he or she still has to do is treating hypertension and becoming more adherent for medication. But this patient has chosen for weight reduction in a first step for cardiovascular risk treatment.* (Interview 1 with project leader)

While project leaders hoped that the implementation of clinical information systems would improve care for those with a chronic illness, the D&I of the system to this point has come at a significant time–cost in both sites. As Wears and Berg noted, while electronic medical records are often thought of as a panacea for the ills of medical documentation, this is often more dream than reality [14]. To meet the goal of including patients into the patient portal of the new record system, additional time on the part of the clinician and of the project leader was needed, time to tinker with the system, to tailor the system to the needs of the clinicians and of the individual patient, who may or may not have had computer access.

#### Project leadership

For this two-practice project, project leadership took the form of a team of two GPs and a nurse manager. The nurse’s duties included interacting directly with staff at the practices, coordinating the research efforts, and aiding the practice staff as they adopted disease management principles. The work of the project leadership team started:

*by organizing meetings. That’s why we start with 4 meetings and why we start at practice level. Speak with the GPs and the nurses. And we have learned to start slowly, go slowly. I will not tell my GPs to start with 100 patients but will tell my GPs we will start very slowly. … But if it works, we have to change the practice. I hope it works. But we have to wait for it still.* (Interview 1 with project leader)

Changing the practice was seen to need to begin slowly in order to gain support from clinicians and staff at the two GP offices. The project leader saw this coordination and background work as necessary before large-scale changes in patient care were implemented. While this work can be seen as an investment, it was likely a notable source of D&I costs.

Another significant challenge for the project leadership team was to work to procure additional funding for the DMP.

*Step by step, we write down [the plan] for the insurance company. Because they will first look at the plan. After they will decide whether to give us some money. And when you are going to visit your GP, the GP will receive 9 Euros. But when you do the visit according to our rules of cardiovascular risk management, we think that the consult will take half an hour so we have asked to the insurance company not 9 Euros but 25 Euros.* (Interview 1 with project leader)

The complete implementation of the program goals cost more time at the patient care level as well as the time and effort invested at the project leader level to procure more funding.

## Discussion

The findings of this study show that large variation exists in the D&I costs of DMPs implemented in the Netherlands. This variation can be explained by the large variability in DMP development duration, size of DMP providing organization, and the level of care in the providing organization prior to the implementation of a DMP. The qualitative analysis showed that these characteristics were associated with the attributes of the interventions, project leadership, and the history of the ICT systems used in a DMP.

The DMP development duration is positively related to the labor intensiveness during the development phase and development costs. Considering that the development costs are highly positively correlated to the implementation costs, the length of the development phase is an important cost driver of D&I costs.

The research on the case studies and other qualitative research conducted in the remaining 19 sites highlighted that the D&I of an ICT system was an involved process. While previous literature shows that a well-developed ICT system is one of the main preconditions of successful implementation of bundled payments and DMPs in the Netherlands [6], the work required to develop and implement ICT systems was, at the sites, time-consuming and costly. In the diabetes case study, the majority of the development work involved in implementing the ICT system occurred before the study period, but nonetheless the work did happen; however, the cost for this work is not included in the financial data in the diabetes project. As the ICT work (and the costs associated) was included in the D&I period of the CVRM project, this may be an explanation for variation in costs. The D&I of adequate ICT is important for all stakeholders in chronic care. It is important for care groups with the aim of achieving lower D&I costs, for health insurers in order to contract chronic care at lower cost, and for public authorities with the purpose of controlling healthcare expenditure by supporting managed care for chronic diseases. As our data shows, this D&I requires time, financial support, and a flexibility of goals, targets, and timelines, no matter when it occurs or how the D&I is funded.

The qualitative research conducted at the sites revealed that the role of the project leader was an important one, with more established projects with experienced project leaders and managers spending less time on the early development of the programs. Project leaders were responsible for guiding the programs, working with clinicians, delegating responsibilities, and developing contacts with outside funders and vendors. In the studied sites, we saw that in projects with a longer history (and with a project leader with more experience in leading healthcare projects and in the DMP project in specific), the relationships needed for smooth, efficient project management were likely developed in the early years of the programs and the costs for these efforts have not been included in the D&I costs (as was seen in diabetes project). In the CVRM project, these relationships were in the process of being developed during the study in an incremental manner through meetings, developing project plans, and the slow introduction of changes. Project leadership, in general, was especially relevant in that organizational and management failure threaten the successful implementation of disease managed care facilitated by bundled payment in the Netherlands [6].

Project leaders had a fluid role and flexibility within the project, as meeting project goals often requires adaptation. Whether by offering new tools online or printing for patients who have limited computer access at home, this constant adaptation by project leaders and clinicians can be seen as “persistent tinkering in a world full of complex ambivalence and shifting tensions” [18]. Through tinkering, project leaders worked to meet the changing needs of patients, of the healthcare system, and of themselves. Yet tinkering was a slow and often invisible process, as was much of the work of project leaders when tailoring interventions, applying for funding, or working with researchers. This tinkering was constant during the study and programs, but as our data shows, appeared to be more prevalent in the D&I stages as the project leaders are working with new vendors, systems, and care plans. This prevalence of tinkering in the early stages of a DMP may have resulted in higher D&I costs.

The specific DMP populations, too, may have had a significant impact on the D&I of the DMPs. The CVRM DMP was working with patients with low SES, many of whom were reported to be complex patients with limited access to resources. Accommodating the needs of this population may have required more tinkering, more effort from project leaders, and more time from clinicians. These characteristics could have played a role in the higher D&I costs for this site.

Our findings also suggest that large organizations providing DMPs are more likely to have lower D&I costs than smaller organizations. This indicates the existence of economies of scope where large organizations may have already established ICT systems, managerial knowledge, and available capital in other care (e.g. public health and prevention) and disease areas that can be also used in the development of disease specific DMPs. This is supported also by the negative relation between the existing level of disease managed care (as measured by the PACIC) in the first phase of implementing a DMP with D&I costs. This unveils existing synergies between projects within organizations. The economies of scope may appeal financially attractive to DMP providers since they might increase the profit margin of providing DMPs for different diseases. The provision of DMPs that could address different disease areas and multi-morbidity could also tackle the criticism of the current DMPs that they are narrowly focused on a single disease while chronic patients need broader care because they often have one or more other diseases (55% of the patients in our sample have more than one chronic disease). Such a development could also tackle the hesitations of health insurers in contracting DMPs.

Moreover, the minor, though negative, relation between the number of DMP participants and the implementation costs, as illustrated in Figure 4, may indicate the presence of economies of scale. The more patients included in a DMP, the lower the marginal costs of implementation. This can be attributed to fixed costs that are divided by more DMP participants. Capital and operating costs, which are included in the implementation costs, are known cost components subject to economies of scale. This financial advantage of large organizations may attract health insurers to purchase DMP from them hoping for a lower bundled payment per DMP participant. However, as in all industries, the number of participants that lowers the marginal costs of DMP implementation should be investigated because further inclusion can lead to higher costs.

Furthermore, we found no evidence of relation between D&I costs and DMP payment method. Similar to case two, many DMPs reported challenges to get additional financing for the provision of a DMP. However, this did not lead in all cases to higher D&I costs. A previous study found a positive relation between additional funding for disease managed care and healthcare utilization costs [19]. Therefore, care groups should be careful in setting the prices of DMPs when negotiating a bundled payment because that price should cover not only the costs of healthcare for the particular disease but also the D&I costs.

There was also no relation found between D&I costs and type of disease addressed by a DMP. That suggests that none of the diseases studied here can be characterized as “cherries” or “lemons” in the chronic care market with respect to D&I costs. This fact may enable the broadening of the scope of diseases that a DMP addresses by making every disease equally financial attractive to care groups.

The 22 DMPs are considered to be representative of the DMPs that have been implemented the last 3 years in the Netherlands because (a) they cover all diseases for which DMPs have been implemented, (b) they include DMPs in primary and/or secondary care (the most common settings for DMPs), (c) they cover a wide variety of diverse regions and geographic areas with different population density and (d) they differ in the attributes of the DMPs put into place and in the structure of multidisciplinary teams [5]. The study population per disease is also representative of the overall disease population in the Netherlands with respect to age and gender.

The findings of this study are relevant to primary care practices in the Netherlands as well as to health policy makers and primary care practices in other European countries that have implemented or are planning to implement DMPs to achieve integration of chronic care. The programs in this study represent a diversity of chronic diseases that can be addressed by DMPs, ranging from common chronic diseases such as diabetes, CVRM, and COPD, to less frequently addressed chronic diseases such as depression, eating disorders, and mental illnesses. The programs, while diverse, had features in common with other DMPs outside of the Netherlands: addressing the issues of chronic illness through coordinated care, through the use of ICT systems, and through the promotion and implementation of self-management education. This research also provides unique insights into the role of project leaders and of the impact of the history of the programs on D&I costs. Policy makers, DMP designers, and primary care practices in the Netherlands and in Europe can explore the possibilities to contain D&I costs at a minimum level by enhancing leadership and ICT in DMPs as well as exploit existing economies of scope and economies of scale in the provision of DMPs.

## Conclusions

The conclusions of this paper can be summarized into:

- There is wide variation in D&I costs of DMPs, which is driven primarily by the duration of the development phase and the labor intensiveness needed to develop and implement a DMP.
- The level of disease managed care in an organization prior to the provision of a DMP is negatively associated with the D&I costs of this DMP.
- Assisting care groups to develop adequate ICT systems for disease managed care is a win-win situation for all stakeholders.
- It is crucial to define the right mix of DMP interventions and target population and to incorporate these mixes in the planning and budgeting of the DMP development phase.
- There are indications of existence of economies of scale and economies of scope, which may reduce D&I costs. Care groups and health insurers should explore the potentials in exploiting them in a mutually benefiting manner.
- The work done before the sites are awarded study funding, especially in relation to ICT systems, saves time and money during the program and study.
- The experience of project leaders may play a fundamental role in the development and early intervention efforts of the DMP.
- Programs with a longer history, more experienced project leadership, previously established ICT systems, and less complex patient populations had lower D&I costs.

## Additional files

Additional file 1
**Background information about the Dutch health care system.**
Additional file 2: Figure S1 This is the relation between development costs and implementation year 1 costs. **Figure S2.** Relation between the development costs and the duration of the development phase (in months). **Figure S3.** Relation between development costs and the number of FTE’s dedicated to the development of each DMP. **Figure S4.** Relation between annualized development costs and total number of FTE’s in the organization. **Figure S5.** Relation between the development costs and PACIC at baseline. File 6: Relation between implementation costs and PACIC at year 1. **Figure S7.** Development costs by type of payment at the development phase. **Figure S8.** Implementation costs by payment method at implementation year 1. **Figure S9.** Development costs by disease category.

## Abbreviations

DMP: Disease management program
DM: Diabetes mellitus
COPD: Chronic obstructive pulmonary disease
CVR: Cardiovascular risk
ICT: Information and communication technology
D&I: Development and implementation
ZonMw: Nederlandse Organisatie voor Gezondheidsonderzoek en Zorginnovatie (Netherlands Organization for Health Research and Development)
WHO: World Health Organization
FTE: Full-time equivalent
PACIC: Patient assessment of chronic illness care
SES: Socio-economic status
GP: General practitioner
