# Supplementary material for: Identifying and explaining the variability in development and implementation costs of disease management programs in the Netherlands
Source: BMC Health Serv Res. 2014 Oct 26;14:518. doi: 10.1186/s12913-014-0518-0 (PMC4210477; doi:10.1186/s12913-014-0518-0)
Supplement: Additional file 1 — Background information about the Dutch health care system. [file 12913_2014_518_MOESM1_ESM.docx]

**Supplementary (online) File 1: Background information about the Dutch health care system**

Undoubtedly the dominant issue in the Dutch health care system at present is the fundamental reform that came into effect in 2006. With the introduction of a single compulsory health insurance scheme, the dual system of public and private insurance for curative care became history. Managed competition for providers and insurers became a major driver in the health care system. This has meant fundamental changes in the roles of patients, insurers, providers and the government. Insurers now negotiate with providers on price and quality and patients choose the provider they prefer and join a health insurance policy which best fits their situation. To allow patients to make these choices, much effort has been made to make information on price and quality available to the public. The role of the national government has changed from directly steering the system to safeguarding the proper functioning of the health markets. With the introduction of market mechanisms in the health care sector and the privatization of former sickness funds, the Dutch system presents an innovative and unique variant of a social health insurance system. Since the stepwise realization of the blueprint of the system has not yet been completed, the health care system in the Netherlands should be characterized as being in transition. Many measures have been taken to move from the old to the new system as smoothly as possible. Financial measures intended to prevent sudden budgetary shocks and payment mechanisms have been (and are) continuously adjusted and optimized. Organizational measures aimed at creating room for all players to become accustomed to their new role in the regulated market. As the system is still a “work in progress”, it is too early to evaluate the effects and the consequences of the new system in terms of accessibility, affordability, efficiency and quality. Dutch primary care, with gatekeeping GPs at its core, is a strong foundation of the health care system. Gatekeeping GPs are a relatively unusual element in social health insurance systems. The strong position of primary care is considered to prevent unnecessary use of more expensive secondary care, and promote consistency and coordination of individual care. It continues to be a policy priority in the Netherlands. The position of the patient in the Netherlands is strongly anchored in several laws concerning their rights, their relation to providers and insurers, access to information, and possibilities to complain in case of maltreatment. In terms of quality and efficiency of the health care system, the Netherlands is, with some notable exceptions (e.g. implementation of innovations such as day surgery and electronic patient records), an average performer when compared to other wealthy countries. It is too early to tell whether efficiency and quality gains will occur as a result of the 2006 reform.

Text from “Schäfer W, Kroneman M, Boerma W, van den Berg M, Westert G, Devillé W

and van Ginneken E. The Netherlands: Health system review. Health Systems

in Transition, 2010; 12(1):1–229.”
