# Supplementary material for: Identifying and explaining the variability in development and implementation costs of disease management programs in the Netherlands
Source: BMC Health Serv Res. 2014 Oct 26;14:518. doi: 10.1186/s12913-014-0518-0 (PMC4210477; doi:10.1186/s12913-014-0518-0)
Supplement: Additional file 2: Figure S1 — This is the relation between development costs and implementation year 1 costs. Figure S2. Relation between the development costs and the duration of the development phase (in months). Figure S3. Relation between development costs and the number of FTE’s dedicated to the development of each DMP. Figure S4. Relation between annualized development costs and total number of FTE’s in the organization. Figure S5. Relation between the development costs and PACIC at baseline. File 6: Relation between implementation costs and PACIC at year 1. Figure S7. Development costs by type of payment at the development phase. Figure S8. Implementation costs by payment method at implementation year 1. Figure S9. Development costs by disease category. [file 12913_2014_518_MOESM2_ESM.docx]

**Supplementary (online) File 2: Additional figures**

Supplementary Figure 1: This is the relation between development costs and implementation year 1 costs.

Supplementary Figure 2: Relation between the development costs and the duration of the development phase (in months).

Supplementary Figure 3: Relation between development costs and the number of FTE’s dedicated to the development of each DMP

Supplementary Figure 4: Relation between annualized development costs and total number of FTE’s in the organization

Supplementary Figure 5: Relation between the development costs and PACIC at baseline

Supplementary File 6: Relation between implementation costs and PACIC at year 1

Supplementary Figure 7: Development costs by type of payment at the development phase

Supplementary Figure 8: Implementation costs by payment method at implementation year 1

Supplementary Figure 9: Development costs by disease category
